# Doppler Evaluation of Foetal Pulmonary Artery in Intrahepatic Cholestasis of Pregnancy: A Prospective Study

**DOI:** 10.34763/jmotherandchild.20263001.d-26-00035

**Published:** 2026-07-18

**Authors:** Sana Fatima, Nidhi Gupta, Abhinav Jain, Aruna Nigam, Sumedha Sharma

**Affiliations:** Department of Obstetrics & Gynaecology, Hamdard Institute of Medical Science and Research & HAHC Hospital, New Delhi, India; Department of Radiology, Hamdard Institute of Medical Science and Research & HAHC Hospital, New Delhi, India

**Keywords:** Intrahepatic cholestasis of pregnancy, Foetal pulmonary artery, Acceleration time, Ejection time, PATET, Bile acids

## Abstract

**Background:**

Intrahepatic cholestasis of pregnancy (ICP) is the most prevalent liver disease specific to pregnancy, with higher incidence of adverse pregnancy outcomes. Keeping this in mind, this study has been framed to compare the changes in foetal pulmonary artery Doppler in pregnancies complicated with ICP with normal pregnancies.

**Materials and methods:**

This prospective study included total of 100 patients as per the inclusion and exclusion criteria. These patients were divided into two groups: the case group (ICP pregnancies) and control group (normal pregnancies). The Doppler parameters were analysed in terms of acceleration time/ejection time (AT/ET) ratio and the maternal and foetal outcome were evaluated.

**Results:**

Significant differences were found in Doppler parameters between the groups, with longer acceleration times (AT), shorter ejection times (ET), and higher PATET ratios in the ICP cases, which signify altered right ventricular outflow parameters, suggesting compromised pulmonary hemodynamics in newborns of ICP-affected pregnancies. These parameters were significantly influenced by the severity of ICP. The AT and ET, which are measures of cardiac function, show weak correlations with the liver function markers (all below 0.23), suggesting minimal direct relationships between these cardiac parameters and liver dysfunction in ICP patients.

**Conclusion:**

The altered Doppler parameters reflect changes in foetal pulmonary vascular resistance and could be indicative of lung immaturity or altered hemodynamics secondary to the toxic effects of elevated maternal bile acid levels.

## Introduction

Intrahepatic cholestasis of pregnancy (ICP) is the most prevalent liver disease that is specific to pregnancy with an incidence of 0.2% to 2%, subject to significant variation based on ethnicity and geographic location [1]. The classic presentation is pruritus limited to the palms and soles, aberrant liver function, and elevated serum bile acid levels in the second and third trimester. The biochemical abnormalities and symptoms subside following delivery. This condition has been linked to a higher incidence of adverse pregnancy outcomes, which is correlated with the serum level of maternal serum bile acids [2]. Ursodeoxycholic acid (UDCA) is a commonly used treatment for intrahepatic cholestasis of pregnancy. However, there are no randomized trials that are sufficiently large to determine whether this drug reduces the risk of adverse perinatal outcomes, so the clinician must make an individualized decision [3].

Recently, pulmonary artery Doppler has been used for predicting the maturation of the foetal lung in cases of pregnancies complicated with Diabetes Mellitus (DM), severe pre-eclampsia or preterm labour/prelabour rupture of membranes (PROM) [4]. By doing this, the foetal prognosis and need of surfactant can be discussed with the relatives at the time of delivery. The role of pulmonary artery doppler in monitoring other medical disorders, such as ICP, is not well-studied. Bile acids can affect the surfactant synthesis as well as be cardiotoxic to the foetus, so this study examines if this non-invasive measurement of pulmonary artery Doppler can aid in monitoring ICP-affected pregnancies and prognosticating the foetal outcome. This led us to frame this study as a comparison of the fetal pulmonary artery Doppler changes between in pregnancies complicated with ICP and normal pregnancies.

## Materials and methods

This prospective case-control study was conducted in the Department of Obstetrics and Gynaecology, Hamdard Institute of Medical Sciences and Research (HIMSR) & HAHC Hospital, New Delhi, over the period of 1.5 years after approval from the institutional ethical committee. The study population included pregnant women with a period of gestation (POG) ≥36 weeks, who were divided into cases (having complaints of itching and raised serum bile acid levels) and controls (pregnant women without itching or elevated bile acid levels). The women having any obstetric or medical disorder apart from ICP were excluded from the study.

**Figure 1.**
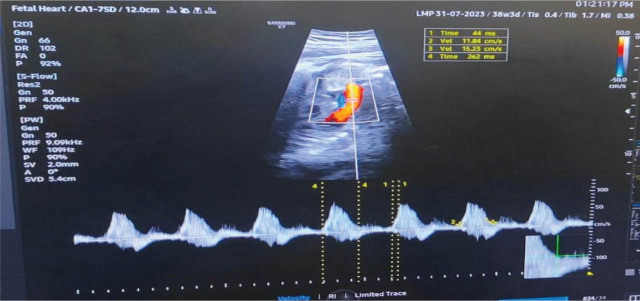
Foetal main pulmonary artery Doppler flow-trace (Ultrasound using 1–5 MHz high resolution convex matric probe, Samsung Hera i10 premium).

All the patients fulfilling the inclusion criteria underwent a pulmonary artery Doppler along with a routine antenatal ultrasound using 1–5 MHz high resolution convex matric probe, Samsung Hera i10 premium ultrasound system. The parameters of the pulmonary artery Doppler were acceleration time (AT), ejection time (ET), and ratio of AT and ET (PATET), and were recorded as shown in Figure 1. Patients were managed according to standard obstetrical management and follow-up examinations were performed until delivery. Foeto-maternal outcome was recorded in the predesigned proforma. Standard statistical analysis was done. A *p*-value of <0.05 was considered statistically significant. Ethical approval was taken from the institute ethical committee (IEC), No. HIMSR/IEC/00112/2025.

## Results

This study included a total of 100 patients as the sample size after fulfilling the inclusion and exclusion criteria. Patients were divided equally among the case group (ICP pregnancies) consisting of 50 patients and the control group (normal pregnancies) consisting of 50 patients. There was no statistically significant difference between both the groups in terms of basic demographic data. The mean age was slightly higher in the case group (28.46 ± 3.59 years) compared to the control group (27.16 ± 4.45 years), but the difference was not statistically significant (t = 1.608, *p* = 0.1111). Table 1 compares the pulmonary artery Doppler parameters of the case and control groups, and shows that the PATET ratio is significantly higher among cases than controls. Comparison of the pulmonary artery Doppler parameters with bile acid values suggests that the Doppler parameters are significantly influenced by the severity of ICP, as shown in Table 2.

**Table 1.** Comparison of pulmonary artery Doppler parameters between cases and controls.

**Pulmonary artery Doppler parameters**	**CASES**		**CONTROL**		**P-VALUE**
	
	**MEAN**	**SD**	**MEAN**	**SD**	
Acceleration time (AT)	47.44	6.56	29.80	8.16	t=8.210
					p<0.0001
Ejection time (ET)	158.06	20.13	172.62	26.24	t=2.148
					p=0.0371
Ratio of Acceleration and Ejection time (PATET)	0.28	0.05	0.17	0.04	t=8.251
					p=0.0001

**Table 2.** Pulmonary artery Doppler parameters according to severity of ICP.

**Pulmonary artery Doppler parameters**	**SEVERITY OF ICP**			**p-VALUE**
	**Mild**	**Moderate**	**Severe**	
AT	46.88 ± 7.63	47.52 ± 4.69	50.33 ± 4.03	F=5.238
				p=0.0063
ET	160.79 ± 25.24	154.14 ± 9.78	171.33 ± 20.17	F=9.890
				p<0.0001
PATET	0.28 ± 0.06	0.28 ± 0.04	0.26 ± 0.02	F=3.571
				p=0.0306

Maternal parameters when compared among cases and controls were not significantly different between the two groups. These parameters included induction of labour (IOL) (case: 10%, control: 4%); mode of delivery: LSCS (case: 56%, control: 48%); vaginal delivery (case: 44%, control: 52%); meconium stained liquor (MSL) (case: 30%, control: 18%); preterm labour (case: 2%, control: 4%); and premature rupture of membranes (PROM) (case: 4%, control: 10%).

**Figure 2.**
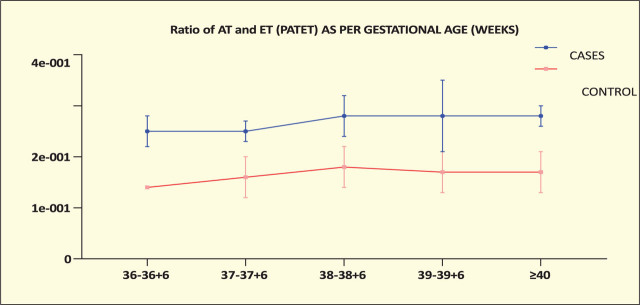
Graphical representations of ratio of AT and ET (PATET) as per gestational age (weeks) between cases and controls.

Comparison of the Doppler values by maternal parameters is tabulated in Table 3. Interestingly, the PATET ratio remained the same in induced and non-induced cases but was marginally lower in induced controls. All comparisons showed statistically significant differences suggesting that induction of labour is associated with measurable changes in right ventricular outflow parameters. In both the case and control group, pulmonary artery values in terms of AT, ET and PATET are not significantly different between the normal delivery patients and patients delivered by LSCS. Mode of delivery may not depend upon the pulmonary artery Doppler values, but there is significant difference among the Doppler parameters between cases and controls, as shown in Table 3. In both groups pulmonary artery Doppler values are not significantly different for meconium stained liquor. That implies that passage of meconium by the foetus may not be related to pulmonary artery Doppler values. There was no significant association between group classification and the occurrence of preterm labour in this study. All the preterm patients underwent spontaneous labour. PATET values according to gestational age are depicted in Figure 2, which shows that in both cases and controls PATET value slightly rises up to 38 weeks, beyond which it plateaus.

**Table 3:** Maternal Parameters.

**IOL**	**CASES**		**CONTROL**		**p-VALUE**
	
	NO^*α^ [n=45]	YES^*β^ [n=5]	NO^*α^ [n=45]	YES^*β^ [n=5]	
AT	46.70±6.43	53.40±3.97	30.00±8.18	25.00±8.49	p<0.0001^*α^
					p<0.0001^*β^
ET	157.20±20.17	166.20±17.21	173.31±26.47	156.00±15.56	p=0.0035^*α^
					p=0.0014^*β^
PATET	0.28±0.05	0.28±0.04	0.17±0.04	0.16±0.04	p<0.0001^*α^
					p<0.0001^*β^

**Mode of Delivery**	NVD^*α^	LSCS^*β^	NVD^*α^	LSCS^*β^	**p-VALUE**

AT	47.86±5.96	47.11±6.87	28.31±6.79	31.42±8.99	<0.0001^*α^
					<0.0001^*β^
ET	157.20±15.5	158.68±2.76	174.65±29.16	170.42±21.81	0.0003^*α^
					0.0003^*β^
PATET	0.27±0.05	0.28±0.06	0.17±0.05	0.18±0.04	<0.0001^*α^
					<0.0001^*β^

**MSL**	Yes^*α^	No^*β^	Yes^*α^	No^*β^	**p-VALUE**

AT	48.93±7.61	48.51±5.62	31.56±7.21	29.41±8.20	<0.0001^*α^
					<0.0001^*β^
ET	163.60±27.67	155.59±14.85	178.44±22.26	171.34±26.55	0.0039^*α^
					0.0004^*β^
PATET	0.25±0.05	0.29±0.05	0.17±0.03	0.17±0.05	<0.0001^*α^
					<0.0001^*β^

**PROM**	Yes^*α^	No^*β^	Yes^*α^	No^*β^	**p-VALUE**

AT	49.50±1.50	47.35±6.61	26.80±5.81	30.13±8.22	<0.0001^*α^
					<0.0001^*β^
ET	138.50±6.50	158.88±19.88	175.20±21.30	172.33±26.4	<0.0001^*α^
					0.0049^*β^
PATET	0.31±0.08	0.27±0.05	0.14±0.03	0.18±0.04	<0.0001^*α^
					<0.0001^*β^

Abruption occurred in 2% of both groups, while antepartum hemorrhage (APH) was seen in 2% of cases but in none of the controls. Postpartum hemorrhage (PPH) was observed in 4% of both groups. Pre-eclampsia was reported in 6% of cases but not in controls, and scar rupture occurred in 2% of controls but not in cases. The comparison of maternal complications between cases (ICP pregnancies) and controls (normal pregnancies) shows no statistically significant differences (*p* = 0.3404).

On comparing the neonatal parameters, the mean birth weight was comparable, with **2991.26 ± 437.08g** in cases and **2953.52 ± 443.74 g** in controls (*p* = 0.7707). The majority of the neonates in both groups had APGAR scores in the range of 8–10 (98% in cases vs. 94% in controls). The incidence of NICU admission is significantly higher in cases (46%) compared to controls (24%) (*p* = 0.0211), showing that the neonates born to mothers with ICP are at a higher risk of requiring intensive care. The incidence of meconium aspiration syndrome is double among controls as cases but this difference is not statistically significant. This indicates that the ICP does not appear to have an impact on the occurrence of meconium aspiration syndrome. The incidence of respiratory distress syndrome (RDS) is significantly higher in cases at 40% compared to controls at 18% (*p* = 0.0153), as shown in Table 3. This indicates a statistically significant association between ICP and an increased risk of RDS in neonates, suggesting that foetuses exposed to ICP are more likely to experience respiratory complications at birth. Table 4 compares the Doppler parameters of neonates and indicates a significant difference in the altered right ventricular outflow parameters between cases and controls, suggesting compromised pulmonary hemodynamics in newborns of ICP-affected pregnancies. The confounding variables like low birth weight or prematurity are comparable in both groups and not significantly different. When Doppler values of above confounding variables were compared, it was shown that they were also not significantly different in foetal weight, weighing <2.5 kg or ≥2.5 kg in both cases and control group.

**Table 4:** Incidence of respiratory distress syndrome (RDS) in neonates among cases and controls.

**RESPIRATORY DISTRESS SYNDROME**	**CASES**		**CONTROL**		**p-VALUE**
	
	N	%	N	%	
No	30	60.00%	41	82.00%	X=5.877
Yes	20	40.00%	9	18.00%	p=0.0153^*^

**Table 5:** Neonatal Parameters.

**Birth Weight**	**CASES**		**CONTROL**		**p-VALUE**
	
	<2500gm^*α^	≥2500gm^*β^	<2500gm^*α^	≥2500gm^*β^	
AT	48.86±5.05	47.21±6.67	27.89±6.71	30.22±8.29	<0.0001^*α^
					<0.0001^*β^
ET	152.86±7.85	158.91±21.14	190.33±41.47	168.73±9.01	<0.0001**α**
					0.0032**β**
PATET	0.28±0.05	0.28±0.05	0.15±0.03	0.18±0.04	<0.0001^*α^
					<0.0001^*β^

**Apgar Score (5 Min)**	4–7^*α^	8–10^*β^	4–7^*α^	8–10^*β^	**p-VALUE**

AT	44.00±0.00	47.51±6.54	29.33±7.93	29.83±8.09	<0.0001^*α^
					<0.0001^*β^
ET	152.00±0.00	158.18±20.11	178.00±14.24	172.28±26.51	<0.0001^*α^
					0.0035^*β^
PATET	0.23±0.00	0.28±0.05	0.17±0.06	0.17±0.04	<0.0001^*α^
					<0.0001^*β^

**NICU ADMISSION**	Yes^*α^	No^*β^	Yes^*α^	No^*β^	**p-VALUE**

AT	46.74±7.55	48.04±5.36	31.92±7.66	29.13±8.09	<0.0001^*α^
					<0.0001^*β^
ET	165.35±24.16	151.85±12.44	174.50±24.31	172.03±26.46	0.0620^*α^
					<0.0001^*β^
PATET	0.26±0.05	0.29±0.05	0.19±0.04	0.17±0.04	<0.0001^*α^
					<0.0001^*β^

**RDS**	Yes^*α^	No^*β^	Yes^*α^	No^*β^	**p-VALUE**

AT	47.50±7.66	47.40±5.58	32.89±8.23	29.12±7.88	<0.0001^*α^
					<0.0001^*β^
ET	161.85±13.13	155.53±23.05	182.11±22.56	170.54±26.21	<0.0001^*α^
					0.0030^*β^
PATET	0.27±0.05	0.28±0.05	0.19±0.05	0.17±0.04	<0.0001^*α^
					<0.0001^*β^

**Figure 3.**
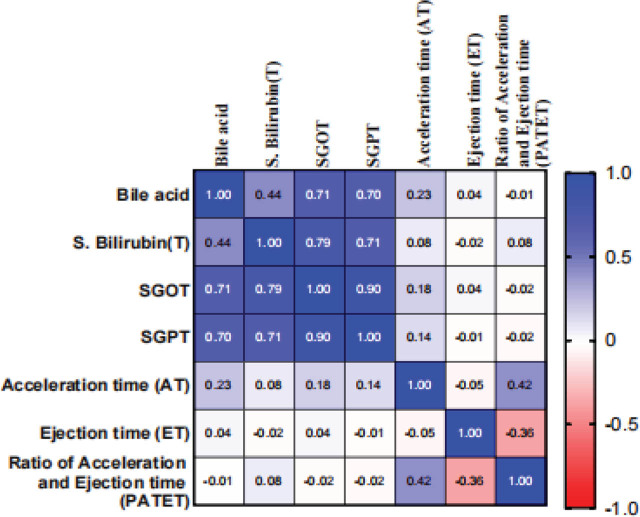
Correlation Matrix in ICP-affected pregnancies.

In patients with ICP, the correlation matrix shows that liver function markers are strongly interrelated (Figure 3). Specifically, bile acid levels are moderately positively correlated with total bilirubin (r = 0.438), and strongly correlated with both SGOT (r = 0.707) and SGPT (r = 0.701), indicating that as bile acids increase, liver enzyme levels also rise, reflecting liver dysfunction. Total bilirubin also has strong correlations with both SGOT (r = 0.789) and SGPT (r = 0.712). On the other hand, the acceleration time (AT) and ejection time (ET), which are measures of cardiac function, show weak correlations with the liver function markers (all below 0.23), suggesting minimal direct relationship between these cardiac parameters and liver dysfunction in ICP patients.

## Discussion

To improve care for pregnancies complicated by ICP, this study looks at these alterations in an effort to find potential markers for early diagnosis of foetal impairment. Foetal impaired oxygenation due to increased bile acids may be indicated by changes in the foetal pulmonary artery Doppler flow patterns. The demographic profile of this study was comparable to that of Yakistiran et al. [5], which found no discernible variations in the socioeconomic position and other demographic traits of mothers. Gupta et al. [6] revealed comparable results, showing a strong positive connection between bile acids and liver enzymes (SGOT, SGPT). Bilirubin did not, however, substantially correlate with either bile acids or liver enzymes, indicating that it would not have a direct impact on Doppler parameters.

Yakistiran et al. [5] found that the there were significant differences between the groups in the mean pulmonary artery AT, ET, PATET, and peak systolic velocity values (*p* = 0.001, *p* = 0.024, and *p* = 0.003, respectively). In their ICP group, mean PATET value was 0.217 ± 0.029, whereas the control group's was 0.180 ± 0.020. Maternal serum bile acid levels were 27.8 ± 16.3 mmol/L on average. In contrast to our findings, Azpurua et al. [7] revealed that the acceleration time/ejection time (AT/ET) ratio was lower (*p*<0.001) and the acceleration time (AT) was considerably shorter in ICP pregnancies. In particular, the AT of their case group was 35 ± 2.4 ms with an AT/ET ratio of 0.18 ± 0.01. Arthius et al. [8] and Zecca et al. [9] noted higher bile acid levels and a significant difference in the rates of admission to the critical care unit. In line with other research, our study group had a higher NICU admission rate.

Kim et al. [10] and Azpurua et al. [7] found a correlation between respiratory distress syndrome (RDS) and lung immaturity and an increased AT/ET ratio. Because ICP cases produce less fetal lung surfactant than healthy babies, this supports the idea that higher PATET in ICP pregnancies may suggest impaired lung development. The ratio of surfactant to albumin has been linked to AT/ET, according to Schenone et al. [11] and Guan et al. [12], who have examined the connection between AT/ET and a later diagnosis of RDS and provided an explanation of this connection using the prenatal lung maturation process. A recent study done by Kumar et al. [13] found that in the neonates who did not develop RDS, the AT/ET ratio showed a mild increasing trend with increasing gestational age (28 weeks onwards), while in the RDS group, it did not increase and showed lower value.

Phuakpoolpol et al. [14] showed statistically significant pulmonary branch diameters and PEDRF correlation with gestational age (*p*<0.001) but at the same time, AT/ET and PI show no correlation with gestational age in their study. Ibrahim et al. [15] suggested in their study that combining the foetal pulmonary artery Doppler indices with two-dimensional ultrasound-derived lung volumetry improves the prediction of neonatal respiratory distress.

Agrawal et al. [16] demonstrated that an AT/ET ratio cutoff value of 0.30 serves as a highly effective non-invasive marker for predicting neonatal RDS. The ratio exhibited a sensitivity of 92.3% and specificity of 91.6%.

Apart from the ICP, foetal pulmonary artery is used for predicting respiratory distress in neonates of various other medical disorders like pre-eclampsia or diabetes. Taha et al. [17] observed that the ROC curve displayed the cut off value of ≤0.25 for the AT/ET ratio and was associated with a sensitivity of 76.92%, a specificity of 100.0%, a PPV of 100.0% and a NPV of 96.7% for prediction of neonatal RDS. Dayanan et al. [18] found that the preeclampsia group exhibited significantly lower PAT/ET ratios (0.180 ± 0.09 vs 0.240 ± 0.07, *p*<.001).

## Conclusion

ICP significantly impacts foetal pulmonary circulation and neonatal outcomes, especially RDS and NICU admissions, despite largely unchanged maternal outcomes. Pulmonary artery Doppler measurements serve as valuable indicators of foetal compromise in ICP and may guide perinatal management strategies. Further large multicentric trails may be needed for cost effectiveness and formulating guidelines.
